# Psychiatric Diagnoses Differ Considerably in Their Associations With Alcohol/Drug-Related Problems Among Adolescents. A Norwegian Population-Based Survey Linked With National Patient Registry Data

**DOI:** 10.3389/fpsyg.2019.01003

**Published:** 2019-05-08

**Authors:** Ove Heradstveit, Jens Christoffer Skogen, Jørn Hetland, Robert Stewart, Mari Hysing

**Affiliations:** ^1^Center for Alcohol and Drug Research, Stavanger University Hospital, Stavanger, Norway; ^2^Regional Centre for Child and Youth Mental Health and Child Welfare, Norwegian Research Centre, Bergen, Norway; ^3^Department of Health Promotion, Norwegian Institute of Public Health, Bergen, Norway; ^4^Department of Psychosocial Science, Faculty of Psychology, University of Bergen, Bergen, Norway; ^5^Institute of Psychiatry, Psychology and Neuroscience, King’s College London, London, United Kingdom; ^6^South London and Maudsley NHS Foundation Trust, London, United Kingdom

**Keywords:** alcohol use, illicit drug use, alcohol/drug-related problems, mental health problems, psychiatric diagnoses, adolescence, registry-based data, population-based sample

## Abstract

The aim of this study was to examine alcohol/drug use and problems across psychiatric diagnoses and to what extent associations between each psychiatric diagnosis and alcohol/drug use and problems were independent from the potential confounding effects of psychiatric comorbidity, socioeconomic status, sex and age. We used a dataset comprising a linkage between a large population-based and cross-sectional study among Norwegian adolescents (the youth@hordaland conducted in 2012) and national registry-based data on specialist mental health care use during the 4 years prior to the survey (2008 to 2011). The study sample included 16 to 19 year olds who participated in the youth@hordaland survey and consented to the linkage with patient registry data (*n* = 9,408). Among these, 853 (9%) had received specialist mental health care and comprised the clinical group, while the rest (*n* = 8,555) comprised the comparison group. The main outcome variables were several self-reported indicators for alcohol/drug use, including any alcohol use, frequent alcohol intoxication, high-level alcohol consumption, and lifetime illicit drug use, as well as one indicator for potential alcohol/drug-related problems: a positive CRAFFT-score. Adolescents receiving specialist mental health care (*n* = 853) reported more frequently alcohol/drug use and problems compared to adolescents not receiving these services (Cohens d’s ranging from 0.09 to 0.29, all *p* ≤ 0.01). Anxiety, depression, conduct disorders, eating disorders, ADHD, and trauma-related disorders were all associated with single measures of alcohol/drug use and problems, with odds ratios (ORs) ranging from 1.58 to 4.63, all *p* < 0.05) in unadjusted models. Trauma-related disorders, depression and conduct disorders were also positively associated with higher scores on a combined indicator of alcohol/drug use and problems (ORs ranging from 1.89 to 3.15, all *p* < 0.01), even after the full adjustment from psychiatric comorbidity and sociodemographic variables (adjusted odds ratios ranging from 1.61 to 2.79, *p* < 0.05). These results suggest that alcohol/drug use and problems were slightly more common among adolescents who received specialist mental health care during the past 4 years compared with the general adolescent population, and adolescents with trauma-related disorders, depression and conduct disorders were high-risk groups for alcohol/drug use and problems.

## Introduction

Mental health problems are prevalent among children and adolescents, with one in five fulfilling criteria for a psychiatric diagnosis (Canino et al., 2004; Belfer, 2008; Merikangas et al., 2010; Barkmann and Schulte-Markwort, 2012). Approximately, 25% of adolescents with a psychiatric diagnosis have at least one additional psychiatric diagnosis (Costello et al., 2003), indicating that psychiatric comorbidity is common. Psychiatric diagnoses are particularly frequent among adolescents with alcohol/drug-related problems, and it is estimated that 37–80% of adolescents with alcohol/drug-related problems have at least one psychiatric diagnosis (e.g., Regier et al., 1990; Rohde et al., 1996; Armstrong and Costello, 2002). Similarly, among adolescents within a psychiatric inpatient setting, one third of the adolescents fulfilled criteria for a substance use disorder (SUD) (Deas-Nesmith et al., 1998).

Previous studies have demonstrated positive associations between a range of psychiatric diagnoses and adolescent alcohol/drug-related problems. Specifically, these findings include anxiety (Kedzior and Laeber, 2014; Stapinski et al., 2016), attention-deficit/hyperactivity disorder (ADHD) (Groenman et al., 2013; Zulauf et al., 2014), eating disorders (Castro-Fornieles et al., 2009; Bisetto et al., 2011), post-traumatic stress disorder (PTSD) (Wolitzky-Taylor et al., 2012; Haller and Chassin, 2014), conduct disorders (Khoddam et al., 2016), depression (Wolitzky-Taylor et al., 2012; Heron et al., 2013; Edlund et al., 2015), and psychotic disorders (Ferdinand et al., 2005; Addington and Addington, 2007). The majority of previous literature has focused on selected associations between single psychiatric diagnoses and symptoms and alcohol/drug-related problems, and few studies have investigated the full range of common psychiatric diagnoses in a single model.

A notable exception is a recent Norwegian study which reported that illicit drug use was four times higher among adolescents receiving psychiatric services compared to the general population (Mangerud et al., 2014). They also reported that depression was the diagnosis associated with the highest frequencies of alcohol and drug use and autism with the lowest; however, participation in the clinical group was low and psychiatric comorbidity was not investigated. Similarly, a study by Wu et al. (2011) investigated associations between a range of psychiatric diagnoses and SUDs. They reported positive associations with SUDs for mood disorders, ADHD, and conduct disorders, while the role of psychiatric comorbidity was not investigated. A study by Boys et al. (2003) on a broad range of psychiatric diagnoses in relation to alcohol/drug-related problems, reported that there was an increased risk of substance use among those with a psychiatric disorder. However, small numbers did not allow differentiation between separate disorders. In sum, there is still limited knowledge on which psychiatric diagnoses are associated with the highest risk for alcohol/drug use and problems among adolescents.

It is also important to consider psychiatric comorbidity in order to clarify whether specificity of risk is indicative of potentially unique psychological mechanisms, or whether “general mental distress” is primarily driving an increased vulnerability for alcohol/drug-related problems. Although some studies have adjusted for psychiatric comorbidity in associations between single psychiatric diagnoses and selected comorbid psychiatric diagnoses (August et al., 2006; Maslowsky and Schulenberg, 2013), the inclusion of psychiatric comorbidity is overall rare. To our knowledge, no previous studies have analyzed associations between a broad range of psychiatric diagnoses and alcohol/drug use and problems among adolescents, while also attempting to adjust these associations from the potential confounding effects of comorbid psychiatric diagnoses. In addition, socioeconomic status (SES), age and gender have been shown to be associated with both mental health and substance use during adolescence (e.g., Van Oers et al., 1999; Schulte et al., 2009), and are as such important co-variates to be considered.

In the present study, we aimed to examine the frequencies of alcohol/drug use and problems among adolescents receiving specialist mental health care compared with a general population of adolescents. In addition, we investigated associations between a broad range of psychiatric diagnoses and alcohol/drug use and problems, while also accounting for the potential confounding effects of other comorbid psychiatric diagnoses, as well as sex, age, and SES. We hypothesized that all psychiatric diagnostic categories – except autism – would be associated with some measure of alcohol/drug use and problems. The lack of similar studies did not allow for specific hypotheses on which diagnoses would have the strongest associations with substance use after the adjustment for psychiatric comorbidity and demographics.

## Materials and Methods

### Study Population

We employed data from the youth@hordaland-survey, which includes information on child and adolescent mental health, lifestyle, school performance and use of health services. Of all 19,430 adolescents born between 1993 and 1995 living in Hordaland County in Western Norway, 10,253 (53%) agreed to participate. The youth@hordaland-survey is a cross-sectional population-based study carried out during early 2012, when the adolescents ranged from 16 to 19 years of age. Participants received information by email and one school hour was used to complete the questionnaires. In addition, adolescents not going to school received the questionnaires by mail at their home address, and mental health services and other institutions were contacted to allow participation for adolescents in these settings. The questionnaires used in the youth@hordaland-survey were web-based.

We linked data from the youth@hordaland-survey to data from the National Patient Registry (NPR) through the participants’ personal identification number. The NPR is the official national registry in Norway on specialist mental health care services, and includes information on specialist mental health care use and Axis 1 psychiatric diagnoses from January 2008 to December 2011, at a time when the adolescents ranged from 12 to 18 years of age, and before youth@hordaland participation. 845 (8.2%) of the adolescents did not provide consent for merging the data from the youth@hordaland-survey with other registries, and were excluded from the analyses. The final sample therefore included 9,408 participants, of whom 853 (9.1%) had at least one registration in NPR.

The Regional Committee for Medical and Health Research Ethics (REC) in Western Norway approved the study. In accordance with the regulations from the REC and Norwegian health authorities, adolescents aged 16 years and older can make decisions regarding their own health (including participation in research). All participants of the study gave written informed consent themselves to participate in the current study. Parents/guardians have the right to be informed, and in the current study, all parents/guardians received written information about the study in advance.

### Measures and Instruments

#### Explanatory Variables: Psychiatric Disorders

The adolescents who had received specialist mental health care (*n* = 853), were assigned to the following diagnostic categories: anxiety (*n* = 132), depression (*n* = 172), conduct disorders (*n* = 32), attention-deficit/hyperactivity disorder (ADHD, *n* = 154), autism spectrum disorders (*n* = 46), eating disorders (*n* = 40), trauma-related disorders (*n* = 66), psychotic disorders (*n* = 10), other diagnoses (*n* = 84), and no Axis 1 psychiatric diagnosis (*n* = 329). 133 adolescents had psychiatric diagnoses from more than one of the specified diagnostic categories, and were assigned to multiple diagnostic categories. Appendix I details the operationalization of the diagnostic categories. Of note, none of the participants who were registered with mental health care use were given a substance-related disorder diagnosis.

Self-reported mental health problems were measured in order to examine differences between individuals that consented (*n* = 9,408) to the linkage between the youth@hordaland-survey and the NPR, and those that did not consent to this linkage (*n* = 845). Specifically, the short version of the Mood and Feelings Questionnaire (SMFQ) (Thapar and McGuffin, 1998) was used to measure symptoms of depression; the SCARED inventory (Birmaher et al., 1999) was used for anxiety symptoms; the Adult ADHD Self-report Scale (ASRS) (Kessler et al., 2007) for symptoms of hyperactivity/inattention; and the Youth Conduct Disorder (YCD) instrument (Lucas et al., 2001) for symptoms of conduct disorders.

#### Outcome Variables: Alcohol/Drug Use and Problems

We constructed a variable for *lifetime alcohol use* based on a single item: ‘Have you ever tried alcohol?’ (Yes/No). Similarly, we constructed a variable for *lifetime illicit drug use* based on a single item: ‘Have you ever tried hash, marijuana or other narcotic substances?’ (Yes/No). We measured *frequent alcohol intoxication* with the question: ‘Have you ever consumed so much alcohol that you were clearly intoxicated (drunk)?’ The original item had five categories ranging from ‘No, never’ to ‘Yes, more than 10 times.’ Frequent alcohol intoxication was defined as drinking so much that one was clearly intoxicated more than 10 times, and on this basis, a dichotomous variable was created. We added up five items that measured how many glasses of (i) beer, (ii) cider, (iii) wine, (iv) spirits, and (v) illegally distilled spirits the adolescents usually consumed during a time period of 14 days. 5,058 adolescents reported any usual alcohol consumption. The *high-level alcohol consumption* variable was defined as the above 90^th^ sex-specific percentile alcohol consumption among the adolescents with any usual alcohol consumption, and a dichotomous variable was created for high-level alcohol consumption (Heradstveit et al., 2017). We also used the six-item, validated CRAFFT scale, in order to indicate adolescents that had a *positive CRAFFT score*. CRAFFT stands for the key words of the six items included in the scale – Car, Relax, Alone, Forget, Friends, Trouble. This scale has been designed to identify potential alcohol-and drug related problems among adolescents and has been demonstrated to have acceptable sensitivity and specificity at a cut-off of ≥2 relative to classifications of problem substance use, substance abuse or substance dependence as identified in structured clinical interviews (Knight et al., 2002; Dhalla et al., 2011). A previous publication based on the youth@hordaland-sample investigated the factor structure and concurrent validity of CRAFFT in relation to self-reported excessive alcohol consumption, frequent binge drinking, and any illicit drug use – and demonstrated a good fit with a single latent construct of alcohol/drug-related problems (Skogen et al., 2013). A dichotomous variable separating those above the cut-off of ≥ 2 on CRAFFT from those below the cut-off were calculated. In our sample the omega internal consistency coefficient (McDonald, 2013) of the CRAFFT scale was 0.88.

Finally, an ordinal variable for level of *risky substance use* was constructed (ranging from 0 to 4), in which we summed up the number of positive scores on lifetime illicit drug use, frequent alcohol intoxication, high-level alcohol consumption, and CRAFFT-scores ≥ 2.

#### Included Co-variates

Age and sex were retrieved from the Norwegian Population Registry, and were available for all participants in the youth@hordaland-sample. In addition, self-reported family financial circumstances was collected as either (1) ‘about the same as others,’ (2) ‘better than others,’ or (3) ‘worse than others.’ Self-reported information on maternal and paternal educational attainment was divided into primary school, high school, or more than 4 years of university or higher education. The variables of self-reported family financial circumstances, paternal educational attainment, and maternal educational attainment were used as a compound measure for SES (Skogen et al., 2014).

### Statistical Analyses

All analyses were performed using STATA V.14.0 (StataCorp, 2015), with the exception that the omega internal consistency coefficient for the CRAFFT questionnaire was calculated in R Core Team (2018). First, frequencies of alcohol/drug use and problems, self-reported symptoms of mental health problems, and sociodemographic variables were examined across individuals who gave their consent to the linkage between the youth@hordaland-survey and the NPR, and those who refused to consent to this linkage (Table 1). Second, the sample was described according to age, sex, SES, and alcohol/drug use and problems among adolescents that had received specialist mental health care services compared to the adolescents that had not receiving these services during the past 4 years (Table 2). Third, psychiatric comorbidity rates within each diagnostic category were described, and all investigated psychiatric diagnoses were analyzed in terms of Spearman’s Rank correlations with other psychiatric diagnoses (Table 3). Fourth, logistic regression models were employed to calculate associations between psychiatric diagnoses received within specialist mental health care and single measures of alcohol/drug use and problems. We then adjusted for the potential confounding effects of comorbid psychiatric diagnoses, in order to determine the independence of associations between each psychiatric diagnosis and substance use. For each psychiatric diagnosis that was investigated, all other psychiatric diagnostic categories were separately entered as potential confounding variables. Finally, we adjusted the analyses for the confounding effects of comorbid psychiatric diagnoses, as well as age, sex and SES (Table 4). Finally, ordered logistic regression models were employed to calculate unadjusted and adjusted odds ratios for the associations between psychiatric diagnoses and the total degree of risky substance use (Table 5). Due to the small size of the psychotic disorders group (*n* = 10), and due to considerable conceptual heterogeneity within the “other diagnoses” group and the “no Axis 1 diagnosis” group, we did not perform analyses of associations between these categories and substance use.

**Table 1:** Frequencies of alcohol/drug use and problems, mental health problems, and sociodemographic characteristics in adolescents excluded from the study sample (*n* = 845), compared with adolescents in the study sample (*n* = 9,408).

	Youth@hordaland-sample (*n* = 10,253)			
	*Study sample:*	*Excluded individuals:*	Cohens d	*p*-Value
	Consented to linkage	Refused consent to linkage		
	with NPR (*n* = 9,408)	with NPR (*n* = 845)		
**Alcohol/drug use and problems**				
Ever tried alcohol, *n* (%)	6,948 (77.2)	573 (77.6)	0.01	0.803
Ever tried drugs, *n* (%)	917 (10.2)	82 (11.1)	0.03	0.436
Frequent alcohol intoxication, *n* (%)	1,766 (18.8)	168 (19.9)	0.03	0.429
High-level alcohol consumption, *n* (%)^1^	495 (5.9)	56 (8.5)	0.11	0.008
CRAFFT ≥ 2, *n* (%)	1,901 (21.2)	161 (21.9)	0.02	0.636
**Mental health problems**				
Anxiety (SCARED), mean (95% CI)	1.51 (1.47, 1.55)	1.55 (1.42, 1.69)	0.02	0.522
Depression (SMFQ), mean (95% CI)	5.88 (5.76, 6.00)	6.30 (5.85, 6.76)	0.07	0.058
Hyperactivity/inattention (ASRS), mean (95% CI)	26.90 (26.68, 27.12)	27.25 (26.39, 28.11)	0.03	0.396
Conduct problems (YCD), mean (95% CI)	0.54 (0.52, 0.57)	0.68 (0.57, 0.79)	0.11	0.004
**Sociodemographic variables**				
Girls, *n* (%)	4,974 (52.9)	425 (50.3)	0.05	0.151
Age, mean (95% CI)	17.4 (17.4, 17.4)	17.6 (17.6, 17.7)	0.26	<0.001
Poor family economy, *n* (%)^2^	650 (7.1)	57 (7.2)	0.00	0.953
Low maternal education, *n* (%)^3^	718 (10.1)	71 (12.8)	0.09	0.050
Low paternal education, *n* (%)^4^	743 (10.7)	55 (10.1)	0.02	0.649

*^*1*^Includes individuals above 90^*th*^ percentile sex-specific alcohol consumption levels in the full youth@hordaland sample (*n* = 10,253).*

*^*2*^Includes individuals reporting family economy as “worse than others.”*

*^*3*^Includes individuals reporting maternal education as restrictred to only primary school.*

*^*4*^Includes individuals reporting paternal education as restrictred to only primary school.*

**Table 2:** Alcohol/drug use and sociodemographic characteristics of the study sample (*n* = 9,408).

	Study sample (*n* = 9,408)			
Demographics	Received specialist mental	Did not receive specialist	Cohens d	*p*-value
	health care services^1^	mental health care services		
	(*n* = 853)	(*n* = 8,555)		
Girls, *n* (%)	499 (58.5)	4,475 (52.3)	0.124	0.001
Age, mean (SD)^2^	17.4 (0.8)	17.4 (0.8)	0.047	0.189
**Family financial circumstances, *n* (%)**			0.173	<0.001
Below average	115 (13.9)	535 (6.4)		–
Average	531 (64.4)	5,623 (67.6)		–
Above average	179 (21.7)	2,165 (26.0)		–
**Mothers education, %^3^**			0.159	<0.001
University/college	258 (43.8)	3,174 (48.9)		–
High school	242 (41.1)	2,687 (41.4)		–
Primary school	89 (15.1)	629 (9.7)		–
**Fathers education, %^4^**			0.174	<0.001
University/college	190 (35.9)	2,770 (43.4)		–
High school	264 (49.8)	2,953 (46.2)		–
Primary school	76 (14.3)	667 (10.4)		–
**Alcohol/drug use and problems**		
Ever used alcohol, *n* (%)	636 (78.0)	6,312 (77.2)	-0.021	0.571
Tried illicit drugs, *n* (%)	148 (18.2)	769 (9.4)	-0.292	<0.001
Frequent drinking to intoxication^5^, *n* (%)	188 (22.0)	1,578 (18.5)	-0.092	0.010
High-level alcohol consumption, *n* (%)	65 (8.7)	442 (5.8)	-0.122	0.001
CRAFFT-score ≥ 2, *n* (%)	233 (28.7)	1,668 (20.4)	-0.202	<0.001
**Total degree of risky substance use**			-0.269	<0.001
No indicators of alcohol/drug use and problems, *n* (%)	419 (56.3)	5,002 (65.4)		–
1 indicator of alcohol/drug use and problem, *n* (%)	136 (18.3)	1,454 (19.0)		–
2 indicators of alcohol/drug use and problems, *n* (%)	103 (13.8)	738 (9.6)		–
3 indicators of alcohol/drug use and problems, *n* (%)	70 (9.4)	375 (4.9)		–
4 indicators of alcohol/drug use and problems, *n* (%)	16 (2.2)	83 (1.1)		–

*CRAFFT: screening scale for identification of potential problematic alcohol and drug use among adolescents.*

*^*1*^Received specialist mental health care services during the 4 years (2008–2011) prior to the youth@hordaland-survey (2012).*

*^*2*^Age at the time that the youth@hordaland-survey was collected.*

*^*3*^Only includes those who with valid response on mother’s education (*n* = 7,079), excluding those having answered that they don’t know (*n* = 2,187).*

*^*4*^Only includes those who with valid response on father’s education (*n* = 6,920), excluding those having answered that they don’t know (*n* = 2,325).*

*^*5*^Above 90^*th*^ percentile alcohol consumption among those adolescents with a present alcohol consumption (*n* = 5,058).*

**Table 3:** Correlation matrix of psychiatric diagnoses (*n* = 853).

	ANX	DEP	COND	ADHD	AUT	EAT	TRA	PSY
ANX (*n* = 132)	–							
DEP (*n* = 172)	**0.246^∗∗∗^**	–						
COND (*n* = 32)	0.001	-0.038	–					
ADHD (*n* = 154)	**-0.091^∗∗^**	-0.038	**0.100^∗^**	–				
AUT (*n* = 46)	-0.059	**-0**.004	0.008	**0.090^∗∗^**	–			
EAT (*n* = 40)	-0.049	**0.096^∗∗^**	-0.044	**-0.090^∗∗^**	-0.028	–		
TRA (*n* = 66)	-0.051	-0.047	0.012	**-0.102^∗∗^**	**-0.069^∗^**	-0.043	–	
PSY (*n* = 10)	**0.074^∗^**	**0.108^∗∗^**	-0.022	-0.023	-0.026	-0.024	-0.032	–

*ANX, anxiety disorders; DEP, depression/mood disorders; COND, conduct disorders; ADHD, attention deficit/hyperactivity disorder; AUT, autism spectrum disorders; EAT, eating disorders; TRA, trauma-related disorders; PSY, psychotic disorders.*

*Bold fonts signify statistically significant associations. ^∗^*p* < 0.05; ^∗∗^*p* < 0.01; ^∗∗∗^*p* < 0.001.*

**Table 4:** Logistic regression analyses of associations between psychiatric diagnoses and alcohol/drug use and problems (*n* = 9,408)^1^.

	High-level alcohol	Frequent alcohol	CRAFFT-score ≥2	Illicit drug use
	consumption	intoxication	OR (95%CI)	OR (95%CI)
	OR (95%CI)	OR (95%CI)		
**Anxiety (*n* = 132)**		
Crude model	1.12 (0.55, 2.32)	0.68 (0.41, 1.11)	1.21 (0.81, 1.83)	**1.97 (1.25, 3.12)**^∗∗^
Adj for comorbidity^2^	0.74 (0.33, 1.64)	**0.48 (0.28, 0.82)**^∗∗^	0.81 (0.51, 1.28)	1.10 (0.65, 1.87)
+Adj for SES+sex+age	1.40 (0.55, 3.57)	0.75 (0.38, 1.49)	0.88 (0.48, 1.60)	1.50 (0.74, 3.01)
**Depression (*n* = 172)**		
Crude model	1.45 (0.83, 2.53)	**1.58 (1.13, 2.23)**^∗∗^	**2.04 (1.48, 2.81)**^∗∗∗^	**2.54 (1.76, 3.69)**^∗∗∗^
Adj for comorbidity^2^	1.42 (0.76, 2.67)	**1.74 (1.18, 2.58)**^∗∗^	**2.14 (1.48, 3.09)**^∗∗∗^	**2.12 (1.36, 3.29)**^∗∗^
+Adj for SES+sex+age	1.02 (0.44, 2.39)	1.31 (0.77, 2.23)	**2.10 (1.30, 3.38)**^∗∗^	1.68 (0.92, 3.08)
**Conduct disorders (*n* = 32)**		
Crude model	1.82 (0.55, 6.05)	1.96 (0.93, 4.14)	**2.48 (1.19, 5.16)**^∗^	**4.63 (2.15, 10.00)**^∗∗∗^
Adj for comorbidity^2^	1.44 (0.41, 5.11)	1.88 (0.86, 4.11)	**2.19 (1.02, 4.71)**^∗^	**3.54 (1.58, 7.92)**^∗∗^
+Adj for SES+sex+age	0.73 (0.08, 6.29)	1.68 (0.51, 5.47)	2.12 (0.69, 6.55)	**6.90 (2.25, 21.17)**^∗∗^
**ADHD (*n* = 154)**		
Crude model	0.85 (0.40, 1.84)	1.32 (0.90, 1.92)	1.09 (0.73, 1.62)	**1.81 (1.16, 2.83)**^∗∗^
Adj for comorbidity^2^	0.74 (0.33, 1.64)	1.30 (0.87, 1.94)	0.92 (0.61, 1.40)	1.37 (0.85, 2.22)
+Adj for SES+sex+age	0.81 (0.28, 2.37)	1.25 (0.71, 2.20)	0.98 (0.56, 1.71)	1.72 (0.93, 3.19)
**Autism disorders (*n* = 46)**		
Crude model	0.82 (0.20, 3.40)	**0.30 (0.09, 0.96)**^∗^	0.49 (0.19, 1.24)	0.43 (0.10, 1.76)
Adj for comorbidity^2^	0.79 (0.18, 3.39)	**0.25 (0.08, 0.81)**^∗^	0.39 (0.15, 1.02)	0.28 (0.07, 1.19)
+Adj for SES+sex+age	0.85 (0.11, 6.73)	**0.28 (0.06, 1.24)**^∗^	0.35 (0.10, 1.25)	0.18 (0.02, 1.41)
**Eating disorders (*n* = 40)**		
Crude model	1.34 (0.41, 4.37)	**2.08 (1.07, 4.03)**^∗^	**2.00 (1.04, 3.84)**^∗^	1.25 (0.49, 3.20)
Adj for comorbidity^2^	1.04 (0.30, 3.63)	1.78 (0.89, 3.56)	1.48 (0.74, 2.93)	0.85 (0.32, 2.26)
+Adj for SES+sex+age	0.96 (0.21, 4.43)	**2.29 (1.01, 5.18)**^∗^	1.20 (0.54, 2.69)	1.06 (0.34, 3.29)
**Trauma-related disorders (*n* = 66)**		
Crude model	**4.60 (2.52, 8.41)**^∗∗∗^	**1.88 (1.11, 3.18)**^∗^	**2.43 (1.25, 4.71)**^∗∗∗^	**2.47 (1.36, 4.48)**^∗∗^
Adj for comorbidity^2^	**4.35 (2.35, 8.04)**^∗∗∗^	**1.76 (1.03, 3.00)**^∗^	**2.43 (1.46, 4.05)**^∗∗^	**2.01 (1.09, 3.72)**^∗^
+Adj for SES+sex+age	**4.77 (2.17, 10.49)**^∗∗∗^	**2.15 (1.06, 4.35)**^∗^	**2.31 (1.20, 4.43)**^∗∗^	2.05 (0.92, 4.54)

*CRAFFT: screening scale for identification of potential problematic alcohol and drug use among adolescents.*

*^*1*^Analyses for psychotic disorders (*n* = 11) and other psychiatric diagnoses (*n* = 84) are not shown in the table.*

*^*2*^For each diagnostic category, psychiatric diagnoses from any other category are included as confounding comorbid diagnoses.*

*Bold fonts signify statistically significant associations. ^∗^*p* < 0.05; ^∗∗^*p* < 0.01; ^∗∗∗^*p* < 0.001.*

**Table 5:** Ordered logistic regression analyses of associations between psychiatric diagnoses and the total degree of risky substance use (*n* = 9,408)^1^.

	Unadjusted model	Adjusted for psychiatric	Adjusted for psychiatric
	OR (95%CI)	comorbidity^2^ AOR (95%CI)	comorbidity^2^ + SES, sex
			and age AOR (95%CI)
Anxiety (*n* = 132)	1.08 (0.74, 1.56)	0.71 (0.48, 1.07)	0.92 (0.54, 1.58)
Depression (*n* = 172)	**1.89 (1.40, 2.55)**^∗∗∗^	**1.93 (1.37, 2.71)**^∗∗∗^	**1.61 (1.03, 2.54)**^∗^
Conduct disorders (*n* = 32)	**3.15 (1.62, 6.12)**^∗∗^	**2.86 (1.43, 5.73)**^∗∗^	**2.79 (1.02, 7.66)**^∗^
ADHD (*n* = 154)	1.34 (0.95, 1.88)	1.20 (0.84, 1.72)	1.44 (0.90, 2.31)
Eating disorders (*n* = 40)	1.81 (0.98, 3.34)	1.33 (0.70, 2.52)	1.34 (0.64, 2.80)
Trauma disorders (*n* = 66)	**2.78 (1.72, 4.48)**^∗∗∗^	**2.48 (1.52, 4.03)**^∗∗∗^	**2.62 (1.37, 5.02)**^∗∗^

*^*1*^Analyses for psychotic disorders (*n* = 11) and other psychiatric diagnoses (*n* = 84) are not conducted. Autism diagnoses (*n* = 46) were also excluded as the proportional odds assumption was not met.*

*^*2*^For each diagnostic category, psychiatric diagnoses from any other category are included as confounding comorbid diagnoses.*

*Bold fonts signify statistically significant associations. ^∗^*p* < 0.05; ^∗∗^*p* < 0.01; ^∗∗∗^*p* < 0.001.*

## Results

For the most part, adolescents who consented to the linkage between the youth@hordaland-survey and the NPR and which therefore constituted the final study sample (*n* = 9,408), were similar to those that did not consent (*n* = 845) (Table 1). However, the individuals that refused consent had somewhat higher frequencies of high-level alcohol consumption (8.5 versus 5.9%, *d* = 0.11, *p* < 0.01), higher mean symptom levels of self-reported conduct problems (0.68 versus 0.54, *d* = 0.11, *p* < 0.01), and were somewhat older (17.6 versus 17.4, *p* < 0.001).

In the study sample (*n* = 9,408), 9.1% (*n* = 853) of the adolescents had received services from Norwegian specialist health care during the past 4 years (2008 to 2011). As outlined in Table 2, adolescents who had received specialist mental health care services were more often female (58.5 versus 52.3%, *p* = 0.001) and of low SES (*d* = 0.17, *p* < 0.001). In addition, adolescents who had received specialist mental health care services had higher frequencies of most alcohol/drug use and problems (d’s ranging from 0.09 to 0.29, all *p* < 0.05) compared with adolescents that had not received specialist mental health care services. The only exception was on the measure for having ever used alcohol, which was non-significant (*p* = 0.571).

Frequencies of comorbidity with other psychiatric diagnoses were examined for each of the included psychiatric diagnoses. Among adolescents who had received a psychiatric diagnosis from a specialist mental health care clinic (*n* = 524), a total of 133 (25.4%) had at least one comorbid psychiatric diagnosis from another diagnostic category (data not shown). Specifically, the prevalence of psychiatric comorbidity were 59.1% (*n* = 78) for anxiety, 62.2% (*n* = 107) for depression, 62.5% (*n* = 20) for conduct disorders, 42.9% (*n* = 66) for ADHD, 60.1% (*n* = 28) for autism, 52.5% (*n* = 21) for eating disorders, 28.8% (*n* = 19) for trauma-related disorders, 70.0% (*n* = 7) for psychotic disorders, and 11.8% (*n* = 39) for “other psychiatric diagnoses.” Table 3 presents the correlation coefficients between the psychiatric diagnoses. Anxiety and depression had the strongest correlation (*r_s_* = 0.246, *p* < 0.001), while all other correlations were either non-significant or had a very small magnitude, spanning from *r_s_* = 0.069 to *r_s_* = 0.108.

In unadjusted models (Table 4), anxiety was associated with illicit drug use (odds ratio [OR] = 1.97, *p* < 0.01); depression was associated with frequent alcohol intoxication (OR = 1.58, *p* < 0.001), a positive CRAFFT score (OR = 2.04, *p* < 0.001) and illicit drug use (OR = 2.54, *p* < 0.01); conduct disorders were associated with a positive CRAFFT score (OR = 2.48, *p* < 0.05) and illicit drug use (OR = 4.63, *p* < 0.001); ADHD was associated with illicit drug use (OR = 1.81, *p* < 0.01); eating disorders were associated with frequent alcohol intoxication (OR = 2.08, *p* < 0.05) and a positive CRAFFT score (OR = 2.00, *p* < 0.05); trauma-related disorders were associated with all alcohol/drug measures (ORs ranging from 1.88 to 4.60, all *p* < 0.05). Additionally, autism was negatively associated with frequent alcohol intoxication (OR = 0.30, *p* < 0.05).

After adjustment for psychiatric comorbidity, anxiety was only negatively associated with frequent alcohol intoxication (adjusted odds ratio [AOR] = 0.48, *p* < 0.01). Depression was still significantly associated with frequent alcohol intoxication, a positive CRAFFT score and illicit drug use (AORs ranging from 1.74 to 2.14, all *p* < 0.05). Conduct disorders were positively associated with a positive CRAFFT score (AOR = 2.19, *p* < 0.05) and illicit drug use (AOR = 3.54, *p* < 0.01). ADHD and eating disorders were no longer associated with any substance use or problems. Autism was still negatively associated with frequent alcohol intoxication (AOR = 0.25, *p* < 0.05). Trauma-related disorders were positively associated with all measures of substance use/problems (AORs ranging from 1.76 to 4.35, all *p* < 0.05).

In fully adjusted models, adjusting for the potential confounding effects of psychiatric comorbidity, SES, sex and age, neither anxiety nor ADHD were significantly associated with any measures of alcohol/drug use and problems. Depression was associated only with a positive CRAFFT score (AOR = 2.10, *p* < 0.01); conduct disorders were associated only with illicit drug use (AOR = 6.90, *p* < 0.01); eating disorders were associated only with frequent alcohol intoxication (AOR = 2.29, *p* < 0.05); and trauma-related disorders were associated with high-level alcohol consumption, frequent alcohol intoxication and a positive CRAFFT-score (AORs ranging from 2.15 to 4.77, all *p* < 0.05). In addition, autism was negatively associated with frequent alcohol intoxication (AOR = 0.28, *p* < 0.05).

Using likelihood-ratio tests of proportionality of odds across response categories, we found only non-significant differences between all the psychiatric diagnoses listed in Table 5 and each ordinal level of indicators for risky substance use in the crude models (*p*-values ranging from 0.27 to 0.93), indicating that the proportional odds assumption underlying the ordered logistic regression models were met (Liao, 1994, p. 41). Autism diagnoses were excluded from this analysis as the proportional odds assumption was not met for this diagnosis.

In unadjusted models employing ordered logistic regression analyses, we found positive associations with the total degree of risky substance use and depression (OR = 1.89, *p* < 0.001), conduct disorders (OR = 3.15, *p* < 0.01), and trauma-related disorders (OR = 2.78, *p* < 0.001). Depression, conduct disorders and trauma-related disorders remained positively associated with the total degree of risky substance use after adjustment for psychiatric comorbidity (AORs ranging from 1.93 to 2.86, *p* < 0.01). Even in fully adjusted models, accounting for the confounding effects of psychiatric comorbidity, SES, sex and age, we found positive associations with the total degree of risky substance use for trauma-related disorders (AOR = 2.53, *p* < 0.01), depression (AOR = 1.61, *p* < 0.05), and conduct disorders (AOR = 2.79, *p* < 0.05).

## Discussion

The present study is to our knowledge the first to compare a broad range of psychiatric diagnoses and their associations with alcohol/drug use and problems during adolescence, while also addressing the role of psychiatric comorbidity. Frequencies of alcohol/drug use and problems were higher among adolescents who had received specialist mental health care services compared to adolescents who had not received such services during the past 4 years; however, the magnitude of these differences was small overall. Importantly, adolescents receiving treatment for a psychiatric disorder differed from controls on all measures of substance use and problems except any alcohol use. This finding may suggest that experimenting with alcohol is normative in adolescence but that excessive use of alcohol, use of other substances, and substance-related negative consequences are occurring at higher rates in those receiving services for psychiatric disorders. Furthermore, the investigated psychiatric diagnoses varied widely in the extent to which they were associated with alcohol/drug use and problems, particularly when the influence of other comorbid psychiatric diagnoses and demographic variables was accounted for.

### Depression and Alcohol/Drug Use and Problems

In unadjusted models, depression was among the psychiatric diagnoses with the most consistent positive associations with alcohol/drug use and problems. Specifically, we found positive associations between depression and all single measures of alcohol/drug use and problems except high-level alcohol consumption. These associations remained significant after adjustment for psychiatric comorbidity. However, the only independent association between depression and alcohol/drug use and problems, after the additional adjustment for sociodemographic factors, was in relation to a positive CRAFFT-score. These findings lend some support to previous studies that have reported positive associations between depression and self-reported adolescent alcohol-related problems (Harrell et al., 2009) and general alcohol/drug use (Crum et al., 2008; Maslowsky and Schulenberg, 2013). Our study adds to previous findings that report positive associations between depression and alcohol intoxication (Crum et al., 2008; Pedersen, 2013), as our findings suggest that the association between depression and frequent alcohol intoxication was significant in the unadjusted model and after the adjustment of psychiatric comorbidity. However, this association was non-significant after the additional adjustment of sociodemographic factors. Moreover, depression was associated with the total degree of risky substance use, and this association was independent from psychiatric comorbidity and sociodemographic factors. In sum, these findings suggest that depression is a robust indicator for the total degree of risky substance use among adolescents.

### Anxiety and Alcohol/Drug Use and Problems

We found that anxiety was positively associated with illicit drug use in unadjusted models. However, this association were non-significant after adjustment for psychiatric comorbidity. This finding suggests that no unique association between anxiety and illicit drug use was present in our data. Interestingly, anxiety was negative associated with frequent alcohol intoxication after adjustment for psychiatric comorbidity, but this association was no longer significant after the additional adjustment for sociodemographic variables. In sum, the present study lends little support to an independent association between anxiety and alcohol/drug use or problems among adolescents, and adjustment of psychiatric comorbidity tended to reduce the positive direction of the associations between anxiety and alcohol/drug use and problems, while the additional adjustment of sociodemographic variables did not affect the estimates in a consistent direction. Importantly, the previous literature is characterized by highly inconsistent findings, pointing to both negative (Schmits et al., 2015, 2016; Nelemans et al., 2016; Savage et al., 2016) and positive associations between anxiety and adolescent alcohol/drug use (Pardee et al., 2014; Birrell et al., 2015; Stapinski et al., 2016). Some studies have suggested that different anxiety disorders (Wu et al., 2010; Ohannessian, 2014) and different anxiety typologies within a given disorder (Tomlinson et al., 2013) yield different prediction of alcohol/drug use and problems, and anxiety may also have a role on adolescent alcohol/drug use and problems through interactions with other diagnoses (Lansford et al., 2008). Future studies should expand the scope to also distinguish between subtypes of anxiety disorders (e.g., social anxiety and generalized anxiety) and their independent associations with alcohol/drug use and problems during adolescence. Also of note, future studies could expand with measures of substance use/problems related to drugs with anxiolytic effects (e.g., alcohol and benzodiazepines), as our study did not include measures of alcohol-problems in particular (as CRAFFT is related to potential alcohol and/or drug-related problems) or benzodiazepine use/problems.

### Autism and Alcohol/Drug Use and Problems

In the present study, autism was negatively associated with frequent alcohol intoxication after the adjustment of both psychiatric comorbidity and sociodemographic variables. In addition, all other single measures of alcohol/drug use and problems tended to go in a negative direction; however, these associations were non-significant. A limited number of previous studies have to our knowledge explored associations between autism and alcohol/drug use and problems among adolescents (Ramos et al., 2013; Mangerud et al., 2014), suggesting a low alcohol/drug use in this group. The present study support these findings by indicating that adolescents with autism had significantly lower odds for frequent alcohol intoxication compared with the general adolescent population. A possible explanation for this finding is that the social skills deficits of autistic adolescents may keep them out of the social situations and/or peer relations typically associated with alcohol consumption in adolescence (Santosh and Mijovic, 2006). However, autism may be associated with alcohol/drug use and problems beyond the adolescent years (Butwicka et al., 2017).

### Eating Disorders and Alcohol/Drug Use and Problems

Our findings suggest that eating disorders were positively associated with frequent alcohol intoxication and a positive CRAFFT-score in unadjusted analyses. However, eating disorders were no longer significantly associated with any alcohol/drug use and problems measures after the adjustment for psychiatric comorbidity, while eating disorders were positively associated with frequent alcohol intoxication after the additional adjustment for demographic variables. This finding supports previous studies linking eating disorders with specific patterns of alcohol use characterized by a loss of control, such as frequent intoxication (Mustelin et al., 2016) and binge drinking (Khaylis et al., 2009). Previous studies indicate that elevated alcohol/drug use and problems are more likely amongst those with bulimia nervosa and binge eating disorders relative to those with anorexia nervosa (e.g., Gadalla and Piran, 2007). The present study did not, however, distinguish between different subtypes of eating disorders, something that should be considered in future research.

### ADHD and Alcohol/Drug Use and Problems

Previous studies have linked both childhood ADHD symptoms (Lee et al., 2011; Heradstveit et al., 2018) and ADHD symptoms during adolescence (August et al., 2006) with adolescent alcohol/drug use and problems. However, several researchers have highlighted that most studies have not controlled for associated psychopathology (August et al., 2006; Lee et al., 2011; Bidwell et al., 2014), therefore leaving doubt on the independence of this association. Our findings were that ADHD was associated with illicit drug use alone; however, this association was non-significant after the adjustment of psychiatric comorbidity as well as after the additional adjustment of sociodemographic variables. These findings suggest that no unique association between ADHD and alcohol/drug use and problems were present in our data, lending support to a study by August et al. (2006). They demonstrated that adolescent ADHD was positively associated with illicit drug use only for individuals with a comorbid externalizing disorder, primarily oppositional defiant disorder. A recent literature review similarly indicated that ADHD does not increase the risk of illicit drug use beyond the effect of conduct-related disorders (Serra-Pinheiro et al., 2013). In sum, our results suggest that ADHD was not independently associated with alcohol/drug use and problems among adolescents, and that comorbid psychiatric disorders may be important confounders in association between ADHD and illicit drug use. However, future studies are encouraged to test whether certain features of ADHD (e.g., impulsivity) might show more unique relations to alcohol/drug use and problems.

### Conduct Disorders and Alcohol/Drug Use and Problems

A range of previous studies have pointed to positive associations between externalizing problems and alcohol/drug use and problems (e.g., Heron et al., 2013; Hopfer et al., 2013; Cerda et al., 2016; Heradstveit et al., 2018; Pedersen et al., 2018), while studies exploring the independence of these associations with respect to comorbidity are more limited. In the present study, we found that conduct disorders were associated with illicit drug use, a positive CRAFFT score, and the total degree of risky substance use in unadjusted models, while we also found independent associations between conduct problems and illicit drug use and the total degree of risky substance use. Hence, conduct disorders appeared to be an important indicator for alcohol/drug use and problems in this study. Also of note, conduct disorders were the only specific psychiatric diagnosis that had an independent association with illicit drug use, highlighting the importance of this diagnosis in relation to illicit drug use among adolescents. This finding lends support to the deviancy proneness model that highlights deviant behaviors as an important risk factor for illicit drug use among adolescents (Tarter et al., 2006).

### Trauma-Related Disorders and Alcohol/Drug Use and Problems

In unadjusted models, trauma-related disorders were positively associated with all single measures of alcohol/drug use and problems, while they were positively associated with all single measures except illicit drug use after the combined adjustment for psychiatric comorbidity and sociodemographic variables. This latter finding suggest that psychiatric comorbidity and sociodemographic variables should be taken into account as potential confounders in the associations between trauma-related disorders and illicit drug use. Additionally, trauma-related disorders were independently associated with the total degree of risky substance use. This finding supports previous studies that report positive associations between trauma-related disorders and alcohol/drug use and problems among adolescents (Giaconia et al., 2000; Haller and Chassin, 2014). The mechanisms behind associations between trauma-related problems and alcohol/drug-related problems are complex. A longitudinal, community-based study by Haller and Chassin (2014) found that PTSD symptoms increased the risk for later alcohol/drug-related problems among adolescents, and the authors argued that these results supported a self-medication hypothesis. However, other mechanisms may potentially be at work. For example, early alcohol/drug-related problems often involves chaotic and violent lifestyles, which could possibly increase the risk for trauma exposure (Deykin and Buka, 1997).

### Strengths and Limitations

The study described here has several strengths. First, the sample consists of a well-defined population-based sample of adolescents aged 16 to 19 years, and was sufficiently large to enable a detailed investigation of the associations of interest. Second, a unique linkage with official registry data on specialist mental health care services was utilized, facilitating an investigation of formal psychiatric diagnoses independently determined according to the ICD-10 by professional mental health practitioners. However, the diagnoses are based on clinical judgment and no inter-rater reliability between the raters exists. Third, we investigated alcohol/drug use and problems across a broad range of diagnostic groups, which is rare in previous research (Mangerud et al., 2014), and enabled us to evaluate the independence of associations with alcohol/drug use and problems across several psychiatric diagnoses. Finally, due to the relatively large sample and comprehensive information about psychiatric diagnoses, we were able to adjust our analyses for psychiatric comorbidity, SES, sex, and age.

Some limitations require consideration when drawing inferences from this study. First, although the measures of psychiatric diagnoses preceded those of alcohol/drug use and problems, the study does not have a stringent longitudinal design, and it is not possible to draw conclusions on the causality between psychiatric diagnoses and alcohol/drug use and problems in this study, since some substance use may have predated the mental health care contacts. Second, the response rate in the population-based sample was 53% and included a relatively low proportion of adolescents with self-reported low SES, who in previous studies are found to have higher levels of mental health problems (e.g., Bøe et al., 2012). Official Norwegian statistics indicate that in 2012, 92% of all adolescents in Norway aged 16 to 18 years of age attended high school, compared with 98% in the youth@hordaland-sample (Hysing et al., 2015). The sample may therefore not have been fully representative of adolescents with psychiatric diagnoses due to selective participation. However, a previous publication from the Bergen Child Study indicated that although non-participation in the survey affected the estimated frequency of mental health problems, it did not affect patterns of associations between sociodemographic characteristics and mental health problems (Heiervang and Goodman, 2011). Nevertheless, our findings on associations between the broad range of psychiatric diagnoses and alcohol/drug use and problems require replication within more comprehensively ascertained clinical samples. Third, we did not differentiate between subtypes of psychiatric diagnoses. Fourth, psychiatric comorbidity was significantly higher within some of the diagnostic categories, particularly anxiety, depression, and conduct disorders. We may therefore have underestimated the independent associations between these psychiatric diagnoses and alcohol/drug use and problems. Fifth, alcohol/drug use and problems were measured by self-report, and does not imply the presence of diagnosable SUDs. In addition, the summed variable of the total degree of risky substance use included both substance use (illicit drug use, high-level alcohol consumption, frequent alcohol intoxication) and substance problems (a positive CRAFFT score). The use of a compound measure can be considered as a limitation, as it is difficult to differentiate between substance use and substance problems. Moreover, this summed variable did not specifically include prescription drug misuse as well as tobacco use. The results may therefore not be generalized to all types of drug use. Sixth, although adolescents that did not consent to the linkage between the youth@hordaland-survey and the NPR were overall similar to those that consented to this linkage, they reported somewhat higher frequency of high-level alcohol consumption, self-reported conduct problems, and higher age. Hence, this limitation may affect the generalizability of our findings. Seventh, the present study included many sets of analyses of associations. Multiple testing might therefore be an issue to consider when interpreting the results. Finally, an important limitation related to the generalizability of the findings from the present study is that individuals with untreated mental health problems in the general youth@hordaland-population were not identified. Psychiatric diagnoses in the present study was restricted to individuals that had received specialist mental health care services during the past 4 years. However, some adolescents in the general population may be suffering from undetected and/or untreated psychiatric disorders, or may have received treatment for psychiatric disorders elsewhere other than through specialist mental health care services (e.g., in private practice or through community-based psychologist services). A range of factors may potentially affect specialist mental health care use, such as functional impairment levels (Hintzpeter et al., 2015) and sociodemographic characteristics (Zwaanswijk et al., 2003). Also, a former wave of the Bergen Child Study concluded that specialist mental health care use differed considerably across psychiatric diagnoses, in which children with emotional disorders were underrepresented in mental health care services (Heiervang et al., 2007). Therefore, our findings on associations between psychiatric diagnoses and alcohol/drug use and problems should be interpreted with caution, particularly in relation to anxiety and depression disorders.

## Conclusion

Alcohol/drug use and problems were slightly more common among adolescents who received specialist mental health care during the past 4 years compared with the general adolescent population. All investigated psychiatric diagnoses – except autism – were associated with some measure of hazardous alcohol/drug use and problems, and adolescents with trauma-related disorders, depression, and conduct disorders had particularly high odds for alcohol/drug use and problems.

## Ethics Statement

The Regional Committee for Medical and Health Research Ethics (REC) in Western Norway approved the study. In accordance with the regulations from the REC and Norwegian health authorities, adolescents aged 16 years and older can make decisions regarding their own health (including participation in research), and thus gave consent themselves to participate in the current study. Parents/guardians have the right to be informed, and in the current study, all parents/guardians received written information about the study in advance.

## Author Contributions

OH has carried out the literature review for the introduction and the discussion section, conducted the statistical analyses, and has written the manuscript. OH, MH, and JS have been involved in the preparation and conduct of the statistical analyses, while all authors (OH, MH, JS, JH, and RS) have reviewed the project and participated in manuscript writing.

## Conflict of Interest Statement

The authors declare that the research was conducted in the absence of any commercial or financial relationships that could be construed as a potential conflict of interest.

## Appendix I

**Table A1:** Full range of ICD-10 psychiatric diagnoses (F and R codes) in the clinical sample, and the psychiatric diagnostic categories employed in the study.

Diagnostic category	ICD-10 code	ICD-10 diagnosis
ADHD	F900	Hyperkinetic disorders
	F901	Disturbance of activity and attention
	F908	Other hyperkinetic disorders
	F909	Attention-deficit hyperactivity disorder, unspecified type
Conduct disorders	F910	Conduct disorder confined to family context
	F911	Unsocialized conduct disorder
	F913	Oppositional defiant disorder
	F918	Other conduct disorders
	F919	Conduct disorder, unspecified
	F920	Depressive conduct disorder
	F928	Other mixed disorders of conduct and emotions
	F929	Mixed disorder of conduct and emotions, unspecified
Anxiety disorders	F401	Social phobias
	F402	Specific (isolated) phobias
	F408	Other phobic anxiety disorders
	F410	Panic disorder (episodic paroxysmal anxiety)
	F411	Generalized anxiety disorder
	F412	Mixed anxiety and depressive disorder
	F413	Other mixed anxiety disorders
	F418	Other specified anxiety disorders
	F419	Anxiety disorder, unspecified
	F420	Predominantly obsessional thoughts or ruminations
	F421	Predominantly compulsive acts (obsessional rituals)
	F422	Mixed obsessional thoughts and acts
	F429	Obsessive-compulsive disorder, unspecified
	F452	Hypochondriacal disorders
	F930	Separation anxiety disorder of childhood
	F931	Phobic anxiety disorder of childhood
	F932	Social anxiety disorder of childhood
	F940	Elective mutism
	F932	Social anxiety disorder of childhood
	F940	Elective mutism
Depression/mood disorders	F310	Bipolar affective disorder, current episode hypomanic
	F311	Bipolar affective disorder, current episode manic without psychotic symptoms
	F313	Bipolar affective disorder, current episode mild or moderate depression
	F316	Bipolar affective disorder, current episode mixed
	F317	Bipolar affective disorder, currently in remission
	F319	Bipolar affective disorder, unspecified
	F320	Mild depressive episode
	F3200	Mild depressive episode
	F321	Moderate depressive episode
	F322	Severe depressive episode without psychotic symptoms
	F328	Other depressive episodes
	F329	Depressive episode, unspecified
	F331	Recurrent depressive disorder, current episode moderate
	F332	Recurrent depressive disorder, current episode severe without psychotic symptoms
	F333	Recurrent depressive disorder, current episode severe with psychotic symptoms
	F338	Other recurrent depressive disorders
	F349	Persistent mood (affective) disorder, unspecified
	F381	Other recurrent mood (affective) disorders
	F412	Mixed anxiety and depressive disorder
Trauma-related disorders	F430	Acute stress reaction
	F431	Post-traumatic stress disorder
	F4320	Adjustment disorder, unspecified
	F4321	Adjustment disorder with depressed mood
	F4322	Adjustment disorder with anxiety
	F4323	Adjustment disorder with mixed anxiety and depressed mood
	F4325	Adjustment disorder with mixed disturbance of emotions and conduct
	F438	Other reactions to severe stress
	F439	Reaction to severe stress, unspecified
Psychotic disorders	F2090	Schizophrenia, unspecified
	F21	Schizotypal disorder
	F231	Acute polymorphic psychotic disorder with symptoms of schizophrenia
	F239	Acute and transient psychotic disorder, unspecified
	F2390	Acute and transient psychotic disorder, unspecified
	F29	Unspecified non-organic psychosis
	F333	Recurrent depressive disorder, current episode severe with psychotic symptoms
Autistic disorders	F840	Childhood autism
	F841	Atypical autism
	F845	Asperger syndrome
	F849	Pervasive developmental disorder, unspecified
Eating disorders	F500	Anorexia nervosa
	F501	Atypical anorexia nervosa
	F502	Bulimia nervosa
	F503	Atypical bulimia nervosa
	F509	Eating disorder, unspecified
Other psychiatric diagnoses	F449	Dissociative (conversion) disorder, unspecified
	F454	Persistent somatoform pain disorder
	F489	Neurotic disorder, unspecified
	F510	Non-organic insomnia
	F512	Non-organic disorder of the sleep-wake schedule
	F54	Psychological and behavioral factors associated with disorders or diseases classified elsewhere
	F601	Schizoid personality disorder
	F633	Trichotillomania
	F640	Transsexualism
	F659	Disorder of sexual preference, unspecified
	F933	Sibling rivalry disorder
	F938	Other childhood emotional disorders
	F939	Childhood emotional disorder, unspecified
	F941	Reactive attachment disorder of childhood
	F942	Disinhibited attachment disorder of childhood
	F951	Chronic motor or vocal tic disorder
	F952	Combined vocal and multiple motor tic disorder (de la Tourette)
	F980	Non-organic enuresis
	F981	Non-organic encopresis
	F988	Other specified behavioral and emotional disorders with onset usually occurring in childhood and adolescence
	F989	Unspecified behavioral and emotional disorders with onset usually occurring in childhood and adolescence
	R418	Other symptoms and signs involving cognitive functions and awareness
	R452	Unhappiness
	R454	Irritability and anger
	R457	State of emotional shock and stress, unspecified
	R458	Other symptoms and signs involving emotional state
	R466	Undue concern and preoccupation with stressful events
Non-diagnosis	1000	No proven diagnosis on Axis I
	1999	Not sufficient information to code on Axis I
	–	No F- or R-codes on Axis I
